# Lower Bird Evenness and Diversity Are Associated With Higher Usutu Prevalence in *Culex pipiens* Mosquitoes

**DOI:** 10.1111/zph.13213

**Published:** 2025-02-18

**Authors:** Victor Rodriguez‐Valencia, Marie‐Marie Olive, Gilbert Le Goff, Marine Faisse, Christophe Paupy, David Roiz

**Affiliations:** ^1^ MIVEGEC, Univ. Montpellier, IRD, CNRS Montpellier France; ^2^ International Joint Laboratory ELDORADO, IRD/UNAM Mérida Mexico; ^3^ ASTRE, Cirad, INRAE. Universite de Montpellier Montpellier France

**Keywords:** Camargue, dilution effect, France, mosquito‐borne diseases, One Health, Usutu

## Abstract

**Introduction:**

The mosquito‐transmitted Usutu virus has spread in the last few years, becoming endemic in several areas of Europe, such as in the southern French region of the Camargue. Our aim was to study the relationships between the presence of the viral agent in *Culex* mosquitoes and the structure of bird communities in the context of the dilution effect.

**Methods:**

We carried out mosquito and bird censuses in several selected localities across a land‐use gradient and screened mosquito pools for flaviviruses. We focused on exploring how host bird diversity, richness, abundance and evenness were associated with Usutu detection in *Cx. pipiens.*

**Results:**

Usutu virus was detected in seven pools of *Cx. pipiens*, and phylogenetic analysis identified Usutu lineage Africa 3, confirming its circulation. The probability of detection in mosquitoes is associated with areas with lower bird evenness and diversity but higher bird abundance and richness and higher *Cx. pipiens* abundances.

**Conclusions:**

Bird evenness was the variable with the greatest explanatory power, being negatively related to the probability of detecting Usutu in *Cx. pipiens*, supporting a dilution effect. These results will help us better understand the relationships between bird community structure and the risk of Usutu mosquito‐borne disease.

**Summary**

- This study explored how bird diversity, richness, abundance and evenness were associated with Usutu virus (USUV) detection in *Culex pipiens* female mosquitoes in Camargue wetlands, France.
- USUV was detected in several *Cx. pipiens* mosquito pools and the phylogenetic analysis identified USUV lineage Africa 3.
- Present results suggested that bird communities characterised by greater evenness and diversity were associated with a lower probability of USUV detection in mosquitoes.
- On the other hand, high bird abundance, richness and *Cx. pipiens* abundance were associated with an increased circulation of USUV in mosquitoes.
- These data enhance current knowledge of the link between biodiversity and mosquito‐borne pathogens.

## 1 Introduction

Mosquito‐borne diseases pose a significant threat to global public health, and with more than 80% of the world's population at risk, they represent a considerable health burden with socio‐economic impacts (Huang et al. 2019; Roiz et al. 2024). Flaviviruses, a diverse group of mosquito‐borne RNA viruses including dengue, Zika, yellow fever and West Nile, are significant human pathogens with considerable public health impacts (Huang et al. 2019). Specifically, *Culex*‐borne zoonotic flaviviruses, such as members of the Japanese encephalitis serogroup—West Nile virus (WNV), Usutu virus, and Saint Louis and Japanese encephalitis viruses—are aetiological agents of neurological diseases (Tolsá‐García et al. 2023).

Usutu virus (USUV), which was isolated in South Africa in 1959, is closely related to the West Nile and Japanese encephalitis viruses (Vazquez et al. 2011; Ashraf et al. 2015; Vilibic‐Cavlek et al. 2020; Tolsá‐García et al. 2023). This virus was first detected in Europe in 1996 in Italy and caused massive blackbird mortality in Austria in 2001 (Angeloni et al. 2023). In subsequent years, USUV was detected in neighbouring countries, where abnormal bird mortality has also been reported (Vazquez et al. 2011; Vilibic‐Cavlek et al. 2020); it has also been detected in mosquitoes, birds, humans and other vertebrates in numerous countries (Angeloni et al. 2023). In 2009, the first two human cases of USUV meningoencephalitis infection in Europe were reported in immunocompromised patients in Italy, although most human cases are asymptomatic (Ashraf et al. 2015). In Europe, USUV antibodies have been found in horses, deer, dogs, wild boars and lizards, although its circulation has gone unnoticed in several regions and countries, such as France (Bournez et al. 2019). USUV has eight distinct lineages, Africa 1–3 and Europe 1–5, suggesting different introductions from Africa and endemic circulation, co‐circulating with West Nile and overlapping with it in geographical distribution, ecological range, host range and vector mosquitoes (Engel et al. 2016).

This virus is maintained in a bird‐mosquito‐bird enzootic cycle, with several avian species serving as reservoirs, for example, *Passer domesticus* , *Pica pica* , *Turdus merula* , *Strix nebulosa* and other members of the Passeriformes (Cadar and Simonin 2022). The transmission cycles are complex and depend on interactions among viruses, hosts, vectors, landscape, climate and human activities (Roiz et al. 2019).

The virus has been detected in various avian species, including the great spotted woodpecker, bullfinch, domestic pigeon, Eurasian blackbird and common kingfisher (Angeloni et al. 2023). The circulation of USUV in Europe, its impact on bird populations and the potential role of different avian species as reservoirs are crucial topics for scientific investigation and public and animal health surveillance within an integrated One Health network (Angeloni et al. 2023).

The Camargue region in the south of France has attracted the attention of arbovirus research. The region's unique ecosystem, characterised by wetlands and a highly diverse avian fauna, is an ideal setting for the amplification and transmission of USUV, with *Culex* mosquitoes, mainly *Culex pipiens* , serving as the primary vectors (Martinet et al. 2023). USUV was first documented in the Camargue region in 2015 (Eiden et al. 2018). However, there is evidence that it has been in circulation in the south of France since 2009, indicating a potential establishment in this ecologically rich area (Cailly et al. 2011; Bournez et al. 2019; Constant et al. 2020). Human Usutu infections with atypical neurological presentation manifested as encephalitis, meningitis and meningoencephalitis in both immunocompetent and immunocompromised patients were reported in the nearby city of Montpellier in 2016 (Simonin et al. 2018). Since humans can be accidental dead‐end hosts, there is a need for integrated vector control and surveillance, an understanding of enzootic cycles, and human surveillance. It also highlights the potential health concerns that USUV poses and the importance of investigating its impact on bird populations and neurotropism (Simonin et al. 2018).

Co‐circulation of USUV and other related arboviruses, such as the West Nile virus (Bahuon et al. 2016), further underlines the importance of public health in studying the dynamics of these pathogens in the Camargue (Zeller and Schuffenecker 2004). Given that no treatments or vaccines for WNV or USUV are available for humans, it is crucial to put in place proper viral prevention and vector control (Constant et al. 2022; Angeloni et al. 2023).

The presence of competent vectors is crucial in transmitting the disease, and in the Camargue, the mosquito species *Culex pipiens* and *Culex modestus* cover this role (Eiden et al. 2018; Soto and Delang 2023). *Culex pipiens* is a significant vector of the Usutu virus in Europe (Tolsá‐García et al. 2023), and its seasonal dynamics in the Camargue have been widely studied (Ponçon et al. 2007). Another essential factor to consider is the presence and composition of vertebrate hosts in the area, particularly birds such as Passeriformes, or species like blackbirds ( *Turdus merula* ) and magpies ( *Pica pica* ), which have a relevant role in the USUV transmission cycle (Nikolay 2015; Clé et al. 2019; Roiz et al. 2019).

The ‘dilution effect’ hypothesis proposes that increasing biodiversity is often associated with a reduction in the risk of infectious diseases (Halliday et al. 2020). It has been suggested that changes in the structure of host communities, rather than biodiversity per se, can explain when a dilution effect should be observed (Halliday et al. 2020). Particularly, high species richness (number of species) and evenness (proportional representation by each species) in vertebrate communities may reduce the risk of human exposure to multi‐host multi‐vector diseases (Ostfeld and Keesing 2000). If the species are not equitably distributed in the community, an increased abundance of competent hosts in relation to non‐competent hosts implies higher disease transmission (Rohr et al. 2020). For multi‐host multi‐vector‐borne diseases in diverse ecological communities, more vector bites of opportunistic vectors could occur on hosts that cannot transmit the pathogen (non‐competent hosts), which dilutes the pathogen in the community (Keesing et al. 2010; Swaddle and Calos 2008). However, the relationship between diversity and risk of zoonotic pathogen transmission might be idiosyncratic, and an amplifying effect has also been observed (Keesing et al. 2006; Salkeld et al. 2013).

The complexity of the interplay between the virus, its ecological reservoirs and the vector and host interactions require a comprehensive, integrated and local approach to understand and mitigate the impact of USUV (Reisen 2010; Lambin et al. 2010; Roiz et al. 2019). Our aim is to explore the associations among bird abundance, species richness, evenness, diversity and vector abundance to the prevalence of Usutu virus in *Culex pipiens* mosquitoes based on field data in the Camargue.

## 2 Materials and Methods

### 2.1 Study Area and Experimental Design

**FIGURE 1:**
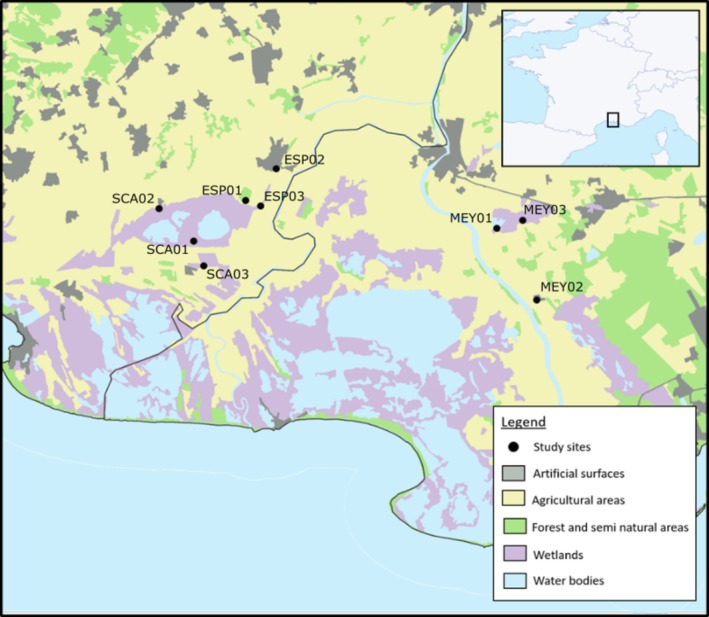
Map showing the study areas in the Camargue region with their land‐use classifications. The collection locations are marked with a black dot; these are distributed across a land‐use gradient ranging from peri‐urban (artificial surfaces) to agricultural (agricultural areas) and natural areas (marshes), with three replicates in each. The characterisation of the land use of the localities has been based on CORINE land cover (CGDD, SOeS 2009). SCA01 is the natural area of Scamandre, SCA02 is the peri‐urban port of Gallician and SCA03 is an agricultural area in Mas Quartet. ESP01 is the natural forest and marshes of Espeyran, ESP02 is the peri‐urban area of Saint‐Gilles, ESP03 is the agricultural area of the field of Espeyran being ESP03B the area of the Château d'Espeyran. MEY01 is the natural area of the Meyranne swamp, MEY02 is the peri‐urban area of Mas Thibert and MEY03 is the agricultural area around Meyranne.

Bordered by the Mediterranean Sea and the two arms of the Rhône River Delta in the South of France, the Camargue region is a highly biodiverse wetland hosting over 300 bird species and more than 1600 invertebrate species. We sampled mosquitoes and took bird censuses during the mosquito season between May and November 2021 in nine locations in three study areas: two study sites in the Gard department (Scamandre [SCA] and Espeyran [ESP], each with three mosquito trapping locations), and one in the Bouches‐du‐Rhône department (Meyrannes [MEY] with three trapping locations) (Figure 1). The characterisation of the land use of the sampling sites has been based on the proportions of each land cover type in a 600‐m‐radius buffer zone extracted from CORINE Land Cover 2012 (CLC 2012) geographic database (https://www.statistiques.developpement‐durable.gouv.fr/corine‐land‐cover‐0), produced by the European earth observation programme Copernicus. Level 1 of CLC 2012 nomenclature was used, including five classes: Artificial surfaces, Agricultural areas, Forest and semi‐natural areas, Wetlands and Water bodies (CGDD, SOeS 2009).

### 2.2 Mosquito Sampling

Adult mosquitoes were captured in Biogent ‘BG‐Sentinel 2’ traps (BGS, Germany) run from a 12 V battery and baited with CO_2_ and BG‐lure. Mosquitoes were captured over 24 h once every 3 weeks at each study site. Besides capturing blood‐fed females, mosquitoes were collected in collection cups inside two resting traps (i.e. 170‐L pop‐up garden bags) (Sauer et al. 2022) placed in each sampling site at a distance of at least 100 m from the BGS, one positioned slightly above the ground (30–50 cm), the other at about 1.5 m from the ground. Resting females were aspirated from the pop‐ups twice per week using a Prokopack model 1419 aspirator (John W. Hock Company, USA), while additional sampling was carried out by aspirating on vegetation in several sessions of 10 min each.

### 2.3 Storage and Identification of Mosquitoes

At the end of each collection session, the catch bags and collection cups were labelled and stored in an ice chest until they were sent to the laboratory, where they were immediately placed in freezers at −20°C until identification the same week. The mosquitoes were then morphologically identified to species using taxonomic identification keys (Schaffner et al. 2001; Becker et al. 2020). Unfed males and females were grouped by date, site and method of capture and stored in pools of up to 50 individuals, with variable size pooling (Gu et al. 2008) with an average of 18 individuals per pool, and were kept at −80°C until viral detection (Roiz et al. 2019). Blood‐fed females were stored individually, and the results of the blood meal identification are presented elsewhere.

### 2.4 Viral Identification

Viral RNA was extracted from pools of up to 50 unfed *Culex pipiens* or *Culex modestus* female mosquitoes with the NucleoSpin RNA Virus kit (Macherey‐Nagel, Germany). The presence of Orthoflavivirus genomes was determined by RT‐PCR (Sánchez‐Seco et al. 2005). Positive Orthoflavivirus pools were confirmed and characterised using a generic RT‐Nested‐PCR with a longer NS5 fragment (Vázquez et al. 2012), followed by a double‐stranded Sanger sequencing of generated amplicons.

### 2.5 Phylogenetic Analysis

The multiple sequence alignment program Geneious 1.6 (https://www.geneious.com) was used to obtain an optimal nucleotide or amino acid sequence alignment file. A consensus of forward and reverse sequences was generated from each sample after alignment to provide the best complementary sequence possible; sequence quality and pairwise identity were verified in each sequence before searching correspondence in BLAST (https://blast.ncbi.nlm.nih.gov/Blast.cgi). Phylograms for an NS5 fragment sequence (Vázquez et al. 2012) were obtained, trimmed and cleaned with this programme. Trees were constructed with Geneious by the Maximum Likelihood method with bootstrap confidence intervals of 1000 heuristic search replicates; the confidence probability of the genetic distance was calculated using a standard error test. A sequence of the Venezuelan equine encephalitis virus was used as an outgroup, and the sequences from GenBank are listed in Table S3.

### 2.6 Bird Census

A professional ornithologist conducted structured bird censuses at each of the three habitats in the three study areas three times (27 in total) during the mosquito season, in July, September and October 2021 to assess the abundance and diversity of avian hosts. Point counts were performed for 6 min at each mosquito trapping location (one at the trap itself and the other four at distances of 150–200 m in each cardinal direction). The counts were made from early morning until 4 h after sunrise within 3 days of mosquito trapping. Only the visual and auditory contacts in front of the observer were counted, as the area behind him was disturbed while he was walking. For each contact, the following variables were recorded: species, number of individuals, distance from the observer and altitude at the moment of the record. Any individuals not observed during the counts were recorded as ‘free observations’ (Bibby 2000). The Shannon diversity index (diversity of species in the community) and bird richness (number of species) were calculated using the ‘BiodiversityR’ and ‘vegan’ packages (Kindt and Coe 2005; Oksanen et al. 2025) in the free R software (R Core team 2023). The evenness (proportional representation by each species) (Ostfeld and Keesing 2000) was also calculated with the Shannon evenness index by dividing the Shannon diversity index by the natural logarithm of richness. These indexes were calculated at a species level.

### 2.7 Statistical Analysis

We used generalised linear models (GLM) to investigate which factors were associated with the probability of finding an Usutu virus infection. The presence or absence of Usutu virus in a locality was included as the dependent variable, while bird abundance, bird richness, bird evenness and bird diversity based on the Shannon diversity index, and vector abundance were included as continuous independent variables. We used GLM with binomial distribution, as the data were overdispersed, and univariate models to avoid collinearity. Model selection was based on the AICc criterion (corrected Akaike information criteria), and the AIC weights were reported as an estimate of the relative support for each model. The explained deviance was calculated as (null model deviance − residual deviance)/null model deviance, and the probability of the presence of the Usutu virus was calculated based on effect size. Statistical analyses were carried out, and figures were created in R version 4. 2. 2 (R Core Team 2023).

## 3 Results

### 3.1 Mosquito Captures

Between May and November 2021, 39,631 mosquitoes (36,163 females and 3468 males) were captured. Fifteen mosquito species were identified, the most abundant species in all the land‐use types being *Culex pipiens* , followed by *Aedes caspius*. Between May and October, 16,149 non‐engorged females of *Cx. pipiens* (14,764) and *Cx modestus* (1385) mosquitoes were captured, accounting for 41% of all the female mosquitoes captured (Table S1).

### 3.2 Bird Census

**FIGURE 2:**
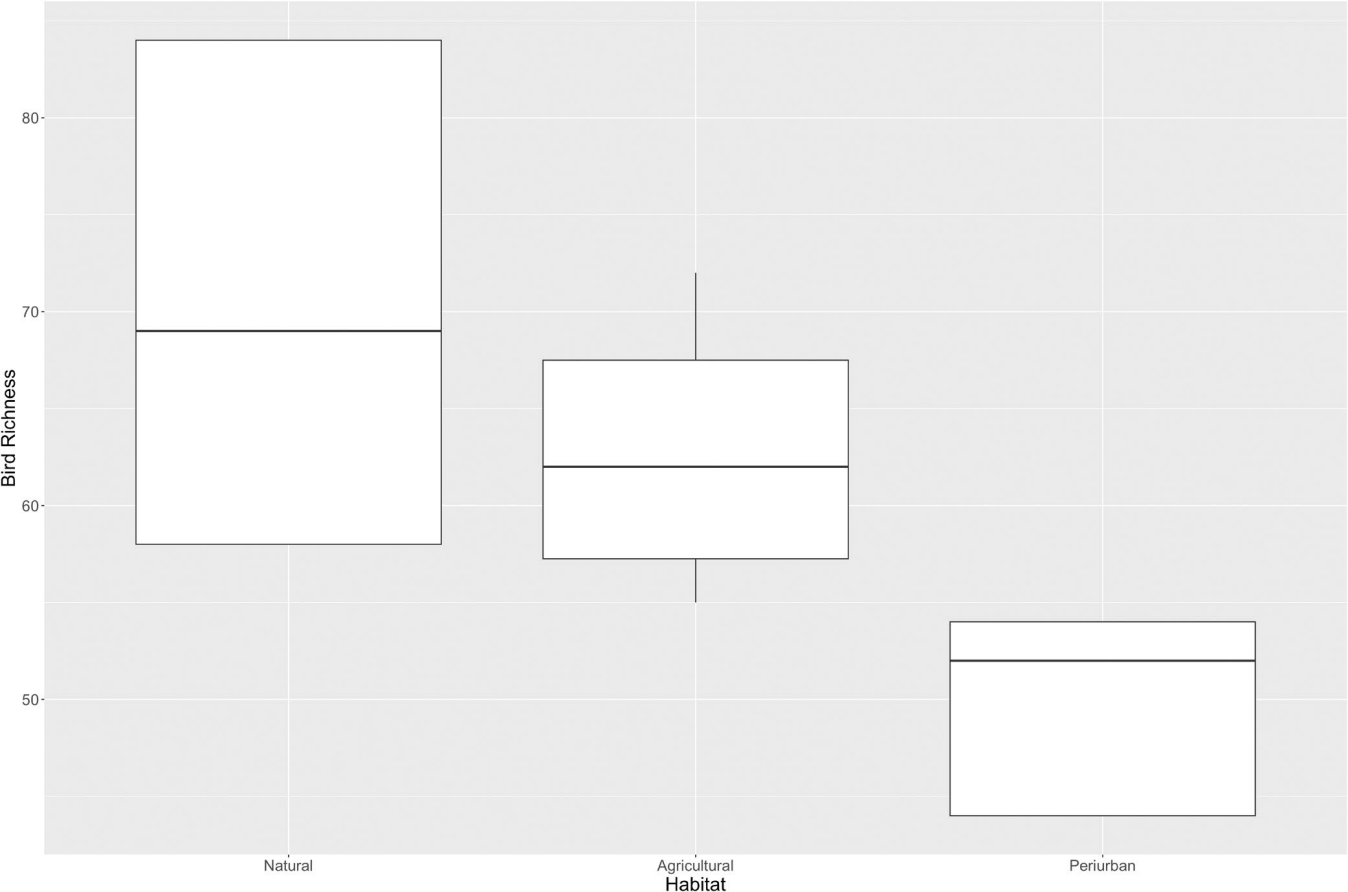
Bird species richness per habitat based on census data.

11,221 individuals belonging to 130 species and 48 avian families were identified. The land‐use type with the highest abundance of birds was the agricultural habitat, with 3379 individual birds of 104 species (Table S2). Approximately, the same number of birds (3166), but the greatest number of species (113), was counted in the natural habitats. Of the three land‐use types surveyed in the census, the peri‐urban habitat had the lowest abundance of birds (1302) and the least species diversity (number of species = 75). Bird richness followed a clear gradient, increasing from the peri‐urban habitats (lowest) to the agricultural habitats (medium) and to the natural areas (highest) (Figure 2).

### 3.3 Viral Identification

**FIGURE 3:**
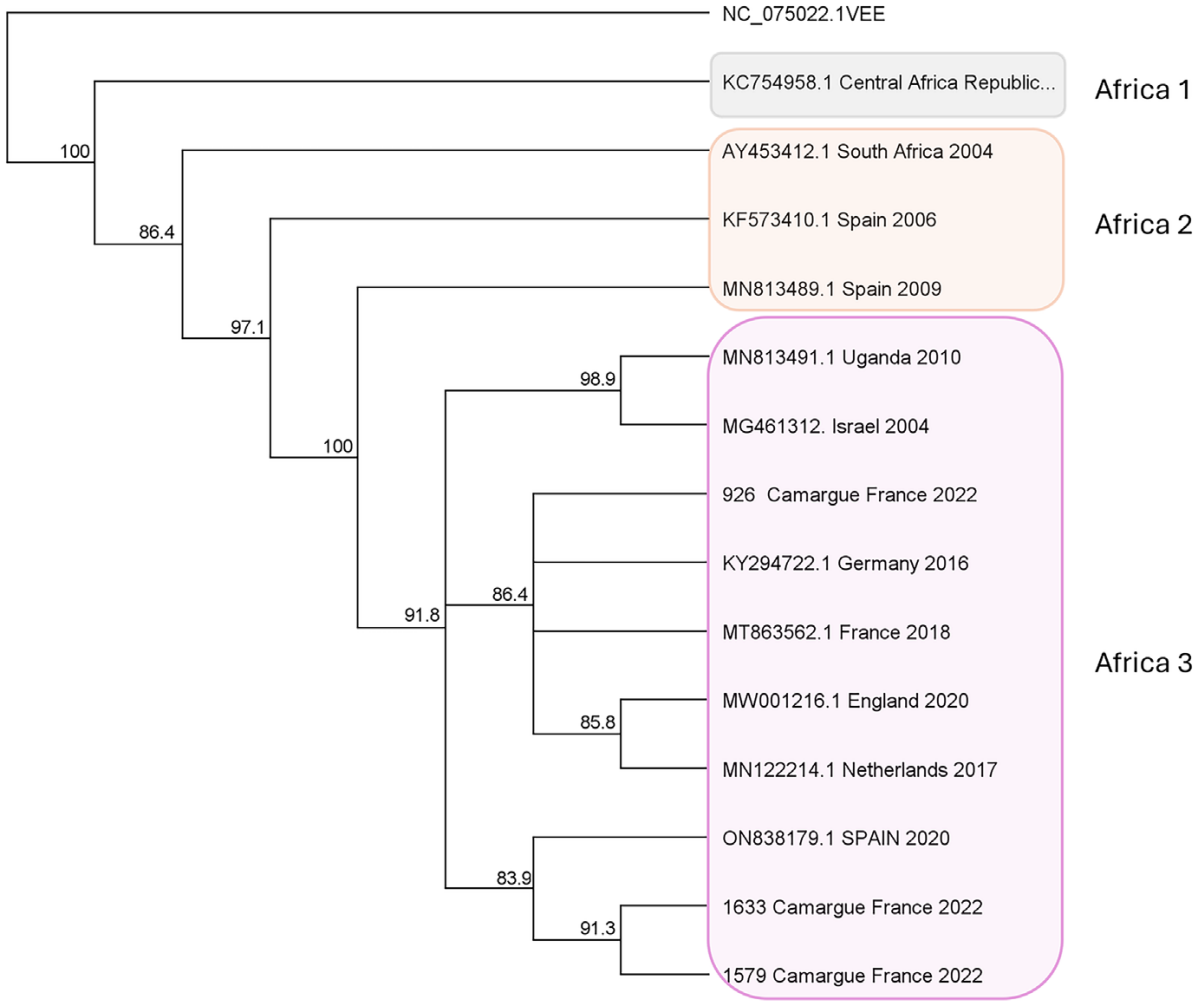
Phylogenetic analysis of USUV sequences. Our positive samples: 926, 1633, 1579 from Camargue, France. EEV: Venezuelan equine encephalitis (outgroup). Usutu sequences used in the phylogenetic study are in Table S3.

We analysed 535 pools of *Cx. pipiens* (*n* = 14,377 females) and 84 pools of *Cx. modestus* (*n* = 1381 females) for the presence of flaviviruses, of which seven *Cx. pipiens* pools were positive for USUV infection (Table 1). Positive pools were identified from the end of July to October; the minimum infection rate increased across the trapping season from 0.03% 95% CI [0.01, 0.07] to 0.21% 95% CI [0.12, 0.32] of infected females per 100 females collected. The infection rate was higher at the collection sites in the natural areas, at 0.21% for every 100 females captured 95% CI [0.16, 0.36]. We made a phylogenetic comparison between known sequences (Table S3) with three of the Usutu sequences identified as belonging to the Africa 3 group (Figure 3).

**TABLE 1:** *Cx. pipiens* pools positive for USUV infection were identified in July, August and October 2021.

Mosquito species	Number of mosquitos	Locality	Habitat	Geographic coordinates	Sampling method	Date
*Culex pipiens*	30	MEY02	Peri‐urban	43°33′17.11′′ N 4°43′34.47′′ E	BG‐Sentinel	28 July
*Culex pipiens*	9	ESP04	Agricultural	43°38′41.9′′ N 4°24′18.5′′ E	Aspiration	10 August
*Culex pipiens*	30	SCA03	Agricultural	43°35′18′′ N 4°20′45′′ E	BG‐Sentinel	18 August
*Culex pipiens*	30	MEY01	Natural	43°36′59.5′′ N 4°40′56.1′′ E	BG‐Sentinel	25 August
*Culex pipiens*	30	MEY03	Agricultural	43°37′20.52′′ N 4°42′40.91′′ E	BG‐Sentinel	25 August
*Culex pipiens*	29	ESP01	Natural	43°38′37.3′′ N 4°23′22.3′′ E	BG‐Sentinel	6 October
*Culex pipiens*	35	SCA01	Natural	43°36′34.2′′ N 4°19′56.6′′ E	BG‐Sentinel	13 October

### 3.4 Usutu Detection, Bird Species and the Dilution Effect

**FIGURE 4:**
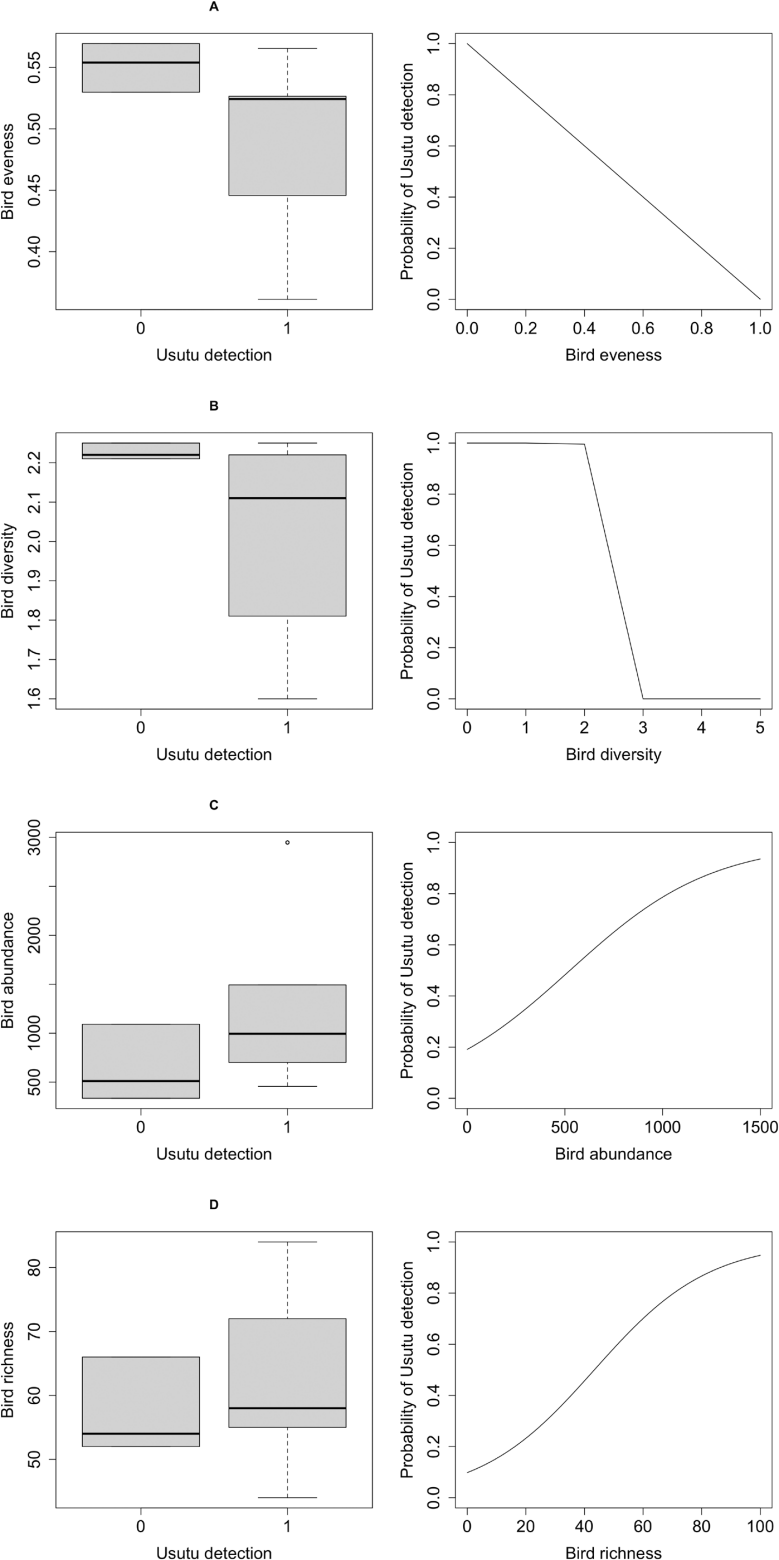
Relationships between (A) bird evenness, (B) diversity (Shannon diversity index), (C) abundance and (D) richness as measured in the census and the probability of detecting *Culex pipiens* infected with Usutu in the Camargue, France.

Bird evenness, selected as the most explanatory variable with 40% of explained deviance and a low AICc, was negatively related to USUV detection in a linear trend (Figure 4A). With a difference of 56 AICc points, bird abundance was chosen as the second informative variable and was positively associated with Usutu detection with a sigmoidal trend (Figure 4C). Bird Shannon diversity index and richness were less explanatory, with negative (Figure 4B) and positive sigmoidal (Figure 4D) relationships, respectively. Finally, vector abundance was the least informative variable for Usutu detection, with only 1% of explained deviance (Table 2). In summary, bird evenness and bird diversity have a relevant negative relationship with the probability of Usutu detection, while bird abundance and richness and vector abundance have a positive relationship with the probability of Usutu detection (Figure 4).

**TABLE 2:** Results of the model selection for the binomial GLM.

Variable	Intercept	Coefficient	LogLik	AICc	Delta	Weight	Explained deviance (%)
Bird evenness	12.84	−24.46	−590.70	1185	0	1,000,000	40
Bird abundance	−3.76	0.005	−618.51	1241	56	< 0.00001	37
Bird diversity (Shannon index)	21.89	−10.07	−709.13	1422	237	< 0.00001	27
Bird richness	−8.83	0.156	−723.31	1451	265	< 0.00001	26
*Cx. pipiens* abundance	0.09	0.0002	−973.92	1952	766	< 0.00001	1

## 4 Discussion

Our study reveals that avian community structure, specifically species evenness and diversity, plays a critical role in modulating the risk of Usutu virus (USUV) detection in mosquitoes. We found that bird communities with higher evenness (balanced distribution of relative species abundances) and greater diversity were associated with a reduced probability of Usutu virus (USUV) detection in *Culex pipiens* mosquitoes. On the other hand, communities with higher bird abundance and species richness, coupled with elevated *Cx. pipiens* abundance, are associated with an increased risk of USUV circulation in mosquitoes. This is the first study highlighting this kind of relationship between bird community structure and USUV, although other studies on WNV in the USA have found similar results (Swaddle and Calos 2008). As these pieces of evidence were correlations and do not imply causation, they should be corroborated by more observations and further studies.

These patterns could contribute to the study of the intrinsic mechanisms of the dilution effect hypothesis, where balanced host communities may buffer pathogen transmission by reducing encounters between competent hosts and vectors (Ostfeld and Keesing 2000; Ostfeld and Keesing 2012; Rohr et al. 2020). Our results highlight that species evenness, rather than species richness data might serve as a potential key indicator for zoonotic risk, being a relevant distinction for future studies testing the biodiversity‐disease associations. Further studies could focus on the local effect of anthropogenic activities on bird community composition/structure, particularly evenness, and the subsequent potential effect on the transmission of a multi‐host disease such as Usutu.

The sustained circulation of USUV in the Camargue region was confirmed through the detection of the Africa 3 viral strain in *Culex pipiens* in 2021, extending reports from 2018 to 2020, underscoring the region as a hotspot for enzootic transmission and spill‐over potential (Eiden et al. 2018; Clé et al. 2019; Constant et al. 2020; Bigeard et al. 2024; Vittecoq et al. 2013; Eiden et al. 2018; Constant et al. 2022). The Camargue necessitates surveillance for viral variants that could be more pathogenic for humans (Clé et al. 2021).

However, the mechanisms that determine enzootic transmission of USUV remain complex (COVARS 2023). Beyond the composition of the avian community, the heterogeneity in vector feeding behaviour and variability in host competence likely modulate transmission dynamics, emphasising the needs of novel integrated metrics that combine host community structure, vector feeding preferences and the variation in host competence (Nikolay 2015; Roiz et al. 2019; Tolsá‐García et al. 2025).

A critical gap is the unresolved role of the secondary vector *Culex modestus* in the Camargue. Despite considerable sampling (84 pools, *n* = 1381 specimens), we were unable to detect Usutu in this species, despite being previously implicated in the transmission in Camargue (Ponçon et al. 2007; Balenghien et al. 2008; Cailly et al. 2011; Soto and Delang 2023). More thorough studies are needed to understand the role of this species in Usutu transmission. Similarly, the role of other arthropods feeding on wild birds, such as ticks, in the transmission of USUV remains unexplored (Bakker et al. 2024). We advocate for innovative and more cost‐effective protocols, such as molecular xenomonitoring that tests excreta shed by trapped mosquitoes for viral RNA (L'Ambert et al. 2023; Bigeard et al. 2024). Finally, more thorough studies are needed to assess the effect of human activities, as land‐use changes, on avian evenness and its impact on multi‐host pathogen dynamics.

## Ethics Statement

Ethical approval is not required for capturing mosquitoes and for observational avian censuses in France. Ethical approval is required only when taking samples with invasive protocols for vertebrates, such as avian species.

## Conflicts of Interest

The authors declare no conflicts of interest.

## Supporting information


Tables S1–S3.

## Data Availability

The data that support the findings of this study are available from the corresponding author upon reasonable request.
