# Supplementary material for: Lower Bird Evenness and Diversity Are Associated With Higher Usutu Prevalence in Culex pipiens Mosquitoes
Source: Zoonoses Public Health. 2025 Feb 18;72(4):359–68. doi: 10.1111/zph.13213 (PMC12016009; doi:10.1111/zph.13213)
Supplement: Supplementary file 1 — Tables S1–S3. [file ZPH-72-359-s001.docx]

| **Mosquito species** | **Periurban** | **Agricultural** | **Natural** | **Total** |
| --- | --- | --- | --- | --- |
| ***Aedes albopictus*** | 126 | 20 | 2 | **148** |
| ***Aedes caspius*** | 4881 | 6457 | 1939 | **13277** |
| ***Aedes detritus*** | 1 | 25 | 0 | **26** |
| ***Aedes sp.*** | 7 | 68 | 12 | **87** |
| ***Aedes vexans*** | 80 | 1238 | 100 | **1418** |
| ***Anopheles hyrcanus*** | 40 | 254 | 126 | **420** |
| ***Anopheles maculipennis*** | 188 | 2591 | 629 | **3408** |
| ***Coquillettidia richiardii*** | 0 | 14 | 13 | **27** |
| ***Culex impudicus*** | 2 | 3 | 11 | **16** |
| ***Culex modestus*** | 268 | 145 | 978 | **1391** |
| ***Culex pipiens*** | 4681 | 3709 | 6417 | **14807** |
| ***Culex sp.*** | 29 | 20 | 112 | **161** |
| ***Culex theileri*** | 293 | 193 | 72 | **558** |
| ***Culiseta annulata*** | 2 | 11 | 1 | **14** |
| ***Culiseta longiareolata*** | 5 | 1 | 0 | **6** |
| ***Culiseta morsitans*** | 0 | 1 | 0 | **1** |
| ***Culicidae*** | 3 | 29 | 4 | **36** |
| ***Uranotaenia unguiculata*** | 0 | 2 | 1 | **3** |
| ***Total*** | **10606** | **14781** | **10417** | **35804** |

**Table S1.** Results of the mosquito capture in all habitats across all seasons.

| **Bird species** | **Periurban** | **Agricultural** | **Natural** | **Total** |
| --- | --- | --- | --- | --- |
| ***Acrocephalus arundinaceus*** | 0 | 4 | 7 | 11 |
| ***Acrocephalus melanopogon*** | 0 | 0 | 2 | 2 |
| ***Acrocephalus scirpaceus*** | 7 | 12 | 33 | 52 |
| ***Actitis hypoleucos*** | 0 | 0 | 9 | 9 |
| ***Aegithalos caudatus*** | 0 | 7 | 37 | 44 |
| ***Alauda arvensis*** | 3 | 19 | 7 | 29 |
| ***Alcedo atthis*** | 8 | 13 | 23 | 44 |
| ***Anas crecca*** | 0 | 182 | 32 | 214 |
| ***Anas platyrhynchos*** | 2 | 494 | 119 | 615 |
| ***Anthus pratensis*** | 4 | 25 | 4 | 33 |
| ***Anthus trivialis*** | 7 | 11 | 8 | 26 |
| ***Apus apus*** | 43 | 185 | 23 | 251 |
| ***Aquila fasciata*** | 0 | 1 | 0 | 1 |
| ***Ardea cinerea*** | 1 | 13 | 36 | 50 |
| ***Ardea purpurea*** | 1 | 9 | 3 | 13 |
| ***Ardeola ralloides*** | 2 | 10 | 4 | 16 |
| ***Athene noctua*** | 0 | 1 | 0 | 1 |
| ***Bubo bubo*** | 0 | 0 | 1 | 1 |
| ***Bubulcus ibis*** | 26 | 56 | 7 | 89 |
| ***Burhinus oedicnemus*** | 0 | 2 | 0 | 2 |
| ***Buteo buteo*** | 2 | 9 | 0 | 11 |
| ***Calidris minuta*** | 0 | 0 | 5 | 5 |
| ***Carduelis carduelis*** | 51 | 259 | 28 | 338 |
| ***Carduelis chloris*** | 2 | 11 | 8 | 21 |
| ***Carduelis spinus*** | 4 | 6 | 16 | 26 |
| ***Casmerodius albus*** | 0 | 3 | 4 | 7 |
| ***Certhia brachydactyla*** | 1 | 14 | 7 | 22 |
| ***Cettia cetti*** | 38 | 85 | 103 | 226 |
| ***Charadrius dubius*** | 0 | 1 | 5 | 6 |
| ***Charadrius hiaticula*** | 0 | 0 | 3 | 3 |
| ***Chlidonias hybrida*** | 0 | 3 | 10 | 13 |
| ***Chlidonias niger*** | 0 | 0 | 1 | 1 |
| ***Chroicocephalus ridibundus*** | 1 | 455 | 105 | 561 |
| ***Ciconia ciconia*** | 88 | 8 | 1 | 97 |
| ***Circus aeruginosus*** | 0 | 2 | 3 | 5 |
| ***Cisticola juncidis*** | 19 | 10 | 8 | 37 |
| ***Coccothraustes coccothraustes*** | 1 | 0 | 0 | 1 |
| ***Columba livia f. domestica*** | 48 | 3 | 4 | 55 |
| ***Columba palumbus*** | 6 | 108 | 554 | 668 |
| ***Coracias garrulus*** | 7 | 19 | 2 | 28 |
| ***Corvus corone*** | 8 | 28 | 15 | 51 |
| ***Corvus frugilegus*** | 0 | 21 | 0 | 21 |
| ***Corvus monedula*** | 24 | 74 | 190 | 288 |
| ***Cuculus canorus*** | 0 | 1 | 0 | 1 |
| ***Cyanistes caeruleus*** | 9 | 30 | 30 | 69 |
| ***Cygnus olor*** | 0 | 7 | 24 | 31 |
| ***Delichon urbicum*** | 24 | 41 | 21 | 86 |
| ***Dendrocopos major*** | 0 | 4 | 10 | 14 |
| ***Dendrocopos minor*** | 3 | 0 | 14 | 17 |
| ***Egretta garzetta*** | 3 | 16 | 52 | 71 |
| ***Emberiza calandra*** | 0 | 14 | 2 | 16 |
| ***Emberiza cirlus*** | 0 | 0 | 2 | 2 |
| ***Emberiza schoeniclus*** | 3 | 5 | 21 | 29 |
| ***Emberiza schoeniclus witherbyi*** | 0 | 0 | 5 | 5 |
| ***Erithacus rubecula*** | 18 | 44 | 34 | 96 |
| ***Falco tinnunculus*** | 1 | 2 | 0 | 3 |
| ***Ficedula hypoleuca*** | 1 | 19 | 18 | 38 |
| ***Fringilla coelebs*** | 41 | 60 | 26 | 127 |
| ***Fringilla montifringilla*** | 3 | 0 | 0 | 3 |
| ***Fulica atra*** | 0 | 0 | 332 | 332 |
| ***Gallinago gallinago*** | 0 | 4 | 1 | 5 |
| ***Gallinula chloropus*** | 9 | 9 | 15 | 33 |
| ***Garrulus glandarius*** | 0 | 3 | 2 | 5 |
| ***Gelochelidon nilotica*** | 1 | 5 | 14 | 20 |
| ***Grus grus*** | 0 | 52 | 1 | 53 |
| ***Himantopus himantopus*** | 0 | 2 | 17 | 19 |
| ***Hippolais polyglotta*** | 1 | 4 | 1 | 6 |
| ***Hirundo rustica*** | 141 | 44 | 118 | 303 |
| ***Hydroprogne caspia*** | 0 | 0 | 2 | 2 |
| ***Larus melanocephalus*** | 4 | 201 | 9 | 214 |
| ***Larus michahellis*** | 7 | 152 | 12 | 171 |
| ***Loxia curvirostra*** | 0 | 0 | 2 | 2 |
| ***Lullula arborea*** | 0 | 1 | 3 | 4 |
| ***Luscinia megarhynchos*** | 7 | 26 | 19 | 52 |
| ***Luscinia svecica*** | 0 | 0 | 2 | 2 |
| ***Merops apiaster*** | 34 | 126 | 52 | 212 |
| ***Milvus migrans*** | 1 | 3 | 3 | 7 |
| ***Motacilla alba*** | 8 | 114 | 5 | 127 |
| ***Motacilla cinerea*** | 4 | 0 | 4 | 8 |
| ***Motacilla flava*** | 4 | 144 | 404 | 552 |
| ***Netta rufina*** | 0 | 14 | 4 | 18 |
| ***Numenius arquata*** | 0 | 3 | 1 | 4 |
| ***Nycticorax nycticorax*** | 0 | 212 | 1372 | 1584 |
| ***Oenanthe oenanthe*** | 0 | 4 | 0 | 4 |
| ***Oriolus oriolus*** | 2 | 1 | 2 | 5 |
| ***Pandion haliaetus*** | 1 | 0 | 2 | 3 |
| ***Panurus biarmicus*** | 0 | 0 | 6 | 6 |
| ***Parus major*** | 15 | 35 | 26 | 76 |
| ***Passer domesticus*** | 160 | 26 | 89 | 275 |
| ***Passer montanus*** | 16 | 0 | 300 | 316 |
| ***Periparus ater*** | 3 | 3 | 0 | 6 |
| ***Phalacrocorax carbo*** | 2 | 28 | 64 | 94 |
| ***Phasianus colchicus*** | 0 | 5 | 0 | 5 |
| ***Philomachus pugnax*** | 0 | 0 | 3 | 3 |
| ***Phoenicopterus roseus*** | 0 | 0 | 116 | 116 |
| ***Phoenicurus ochruros*** | 12 | 4 | 3 | 19 |
| ***Phoenicurus phoenicurus*** | 0 | 0 | 1 | 1 |
| ***Phylloscopus collybita*** | 8 | 11 | 15 | 34 |
| ***Phylloscopus trochilus*** | 0 | 2 | 3 | 5 |
| ***Pica pica*** | 25 | 7 | 11 | 43 |
| ***Picus viridis*** | 1 | 9 | 7 | 17 |
| ***Platalea leucorodia*** | 0 | 0 | 12 | 12 |
| ***Plegadis falcinellus*** | 0 | 213 | 77 | 290 |
| ***Porphyrio porphyrio*** | 6 | 3 | 43 | 52 |
| ***Prunella modularis*** | 0 | 9 | 10 | 19 |
| ***Rallus aquaticus*** | 1 | 11 | 16 | 28 |
| ***Regulus ignicapilla*** | 1 | 0 | 1 | 2 |
| ***Remiz pendulinus*** | 1 | 6 | 2 | 9 |
| ***Riparia riparia*** | 49 | 10 | 94 | 153 |
| ***Saxicola rubicola*** | 0 | 10 | 0 | 10 |
| ***Serinus serinus*** | 2 | 7 | 1 | 10 |
| ***Sterna hirundo*** | 0 | 2 | 7 | 9 |
| ***Streptopelia decaocto*** | 121 | 45 | 10 | 176 |
| ***Streptopelia turtur*** | 0 | 4 | 12 | 16 |
| ***Strix aluco*** | 0 | 1 | 0 | 1 |
| ***Sturnus vulgaris*** | 135 | 345 | 2057 | 2537 |
| ***Sylvia atricapilla*** | 18 | 55 | 40 | 113 |
| ***Sylvia cantillans*** | 0 | 1 | 1 | 2 |
| ***Sylvia melanocephala*** | 17 | 14 | 16 | 47 |
| ***Tachybaptus ruficollis*** | 1 | 0 | 6 | 7 |
| ***Tetrax tetrax*** | 3 | 0 | 0 | 3 |
| ***Tringa glareola*** | 3 | 9 | 6 | 18 |
| ***Tringa nebularia*** | 0 | 2 | 5 | 7 |
| ***Tringa ochropus*** | 4 | 3 | 11 | 18 |
| ***Tringa totanus*** | 0 | 1 | 0 | 1 |
| ***Troglodytes troglodytes*** | 3 | 7 | 8 | 18 |
| ***Turdus merula*** | 0 | 27 | 20 | 47 |
| ***Turdus philomelos*** | 2 | 18 | 22 | 42 |
| ***Upupa epops*** | 0 | 3 | 1 | 4 |
| ***Vanellus vanellus*** | 0 | 1 | 0 | 1 |
| ***Total*** | **1343** | **4467** | **7207** | **13017** |

**Table S2.** Results of the bird censuses in all habitats across all seasons.

| GenBank ID | Country of detection | Agent | Host | Year of submision | Reference |
| --- | --- | --- | --- | --- | --- |
| NC_075022.1 | USA | Venezuelan equine encephalitis | *Equus asinus* | 2023 | Kinney et al, 1989 |
| KC754958.1 | Central Africa Republic | Usutu Virus Africa 1 | *Cx. perfuscus* | 2013 | Nikolay et al. 2013 |
| AY453412.1 | South Africa | Usutu Virus Africa 2 | *Culex neavei* | 2004 | Bakonyi et al, 2004 |
| KF573410.1 | Spain | Usutu Virus Africa 2 | *Cx. pipiens* | 2008 | Busquets et al. 2008 |
| MN813489.1 | Spain | Usutu Virus Africa 2 | *Culex perexiguus* | 2009 | Kuchinsky  Et al 2020 |
| MN813491.1 | Uganda | Usutu Virus Africa 3 | *Culex sp.* | 2010 | Kuchinsky  Et al 2020 |
| MG461310.1 | Israel | Usutu Virus Africa 3 | *Culex perexiguus* | 2018 | Lustig et al 2018 |
| KY294722.1 | Germany | Usutu Virus Africa 3 | *Turdus merula* | 2016 | Ziegler et al. 2018 |
| MT863562.1 | France | Usutu Virus Africa 3 | *Turdus merula* | 2018 | Cle et al, 2020 |
| MW0012161 | England | Usutu Virus Africa 3 | *Turdus merula* | 2020 | Follyet al 2020 |
| MN122214.1 | Netherlands | Usutu Virus Africa 3 | *Turdus merula* | 2017 | Oude Munnink et al 2020 |
| ON838179.1 | Spain | Usutu Virus Africa 3 | *Cx. pipiens* | 2020 | Vazquez et al, 2022 |

**Table S3.** Sequences of Usutu used for the phylogenetic analysis. Venezuelan equine encephalitis was used as in outgroup.

**References**

Bakonyi, T., Gould, E. A., Kolodziejek, J., Weissenbock, H. and Nowotny, N. (2004). Complete genome analysis and molecular characterization of Usutu virus that emerged in Austria in 2001: comparison with the South African strain SAAR-1776 and other flaviviruses. Virology 328 (2), 301-310.

Busquets, N., Alba, A., Allepuz, A., Aranda, C. and Ignacio Nunez, J. (2008). Usutu virus sequences in Culex pipiens (Diptera: Culicidae), Spain Emerging Infect. Dis. 14 (5), 861-863.

Cle, M., Constant, O., Barthelemy, J., Desmetz, C., Martin, M.F., Lapeyre, L., Cadar, D., Savini, G., Teodori, L., Monaco, F., Schmidt-Chanasit, J., Saiz, J.-C., Gonzales, G., Lecollinet, S., Beck, C., Gosselet, F., Foulongne,V., Salinas, S. and Simonin, Y. (2020). Direct Submission Submitted (07-AUG-2020) Animal Health Laboratory, Maisons-Alfort. ANSES, 14 rue Pierre et Marie Curie, Maisons-Alfort 94703, France

Folly, A. J., Lawson, B., Lean, F. Z., McCracken, F., Spiro, S., John, S. K., Heaver, J. P., Seilern-Moy, K., Masters, N., Hernandez-Triana, L. M., Phipps, L. P., Nunez, A., Fooks, A. R., Cunningham, A. A., Johnson, N. and McElhinney, L. M. (2020). Detection of Usutu virus infection in wild birds in the United Kingdom, 2020. Euro Surveillance. 25 (41).

Kinney, R. M., Johnson, B.J., Welch, J.B., Tsuchiya, K.R. and Trent, D.W. (1989). The full-length nucleotide sequences of the virulent Trinidad donkey strain of Venezuelan equine encephalitis virus and its attenuated vaccine derivative, strain TC-83. Virology. 170 (1), 19-30.

Kuchinsky, S.C., Hawks, S.A., Mossel, E.C., Coutermarsh-Ott, S. and Duggal, N.K. (2020). Differential pathogenesis of Usutu virus isolates in mice PLoS Neglected Tropical Diseases. 14 (10), e0008765.

Lustig, Y., Mannasse, B., Orshan, L., Schmidt-Chanasit, J. and Cadar, D. (2017). Continued evolution of Usutu virus. Submitted (08-NOV-2017) Central Virology Laboratory, Ministry of Health, Sheba Way, Ramat-Gan 5262100, Israel

Nikolay, B., Dupressoir, A., Firth, C., Faye, O., Boye, C.S., Diallo, M., Sall, A. A.(2013). Comparative full length genome sequence analysis of Usutu virus isolates from Africa Virology. J. 10 (1), 217.

Oude Munnink, B.B., Munger, E., Nieuwenhuijse, D.F., Kohl, R., van der Linden, A., Schapendonk, C.M.E., van der Jeugd, H., Kik, M., Rijks, J.M., Reusken, C.B.E.M., Koopmans, M. (2020). Genomic monitoring to understand the emergence and spread of Usutu virus in the Netherlands, 2016-2018 10 (1), 2798.

Vazquez, A. and Sanchez-Seco, M. P. (2022). Direct Submission Submitted (24-JUN-2022) National Center of Microbiology, Instituto de Salud Carlos III, Carretera Pozuelo-Majadahonda Km2, Majadahonda, Madrid 28220, Spain

Ziegler, U., Keller, M., Konrath, A., Hoeper, D. and Groschup, M.H. (2016). Direct Submission Submitted (02-DEC-2016) Institute of Novel and Emerging Infectious Diseases, Friedrich-Loeffler-Institut, Federal Research Institute for Animal Health, Suedufer 10, Greifswald - Isle of Riems, Mecklenburg-West Pomerania 17493, Germany
